# The relationship between clinical characteristics and magnetic resonance imaging results of Ménière disease: a prospective study

**DOI:** 10.1038/s41598-021-86589-1

**Published:** 2021-03-30

**Authors:** Wen Xie, Ting Shu, Jiali Liu, Haisen Peng, Niki Karpeta, Pedro Marques, Yuehui Liu, Maoli Duan

**Affiliations:** 1 Department of Otolaryngology, Head and Neck Surgery, The Second Affiliated Hospital of Nanchang University, Nanchang, 330006 China; 2 Department of Otolaryngology, Head and Neck Surgery & Audiology and Neurotology, Karolinska University Hospital, Karolinska Institute, Stockholm, Sweden; 3 Department of Clinical Science, Intervention and Technology, ENT, Karolinska Institute, Karolinska University Hospital, 17176 Stockholm, Sweden; 4 Medical Imaging Center, The Second Affiliated Hospital of Nanchang University, Nanchang, China; 5 Department of Otorhinolaryngology, S. João University Hospital Centre, Porto, Portugal; 6 Unit of Otorhinolaryngology, Department of Surgery and Physiology, University of Porto Medical School, Porto, Portugal

**Keywords:** Clinical trials, Neurological disorders

## Abstract

Ménière disease (MD) is an idiopathic inner ear disorder, and endolymphatic hydrops (EH) being considered to be its pathological basis. Currently, there is no gold standard for diagnosing MD. Previous study has reported visualized EH using MRI by intratympanic gadolinium-based contrast media (GBCM) administration (IT-Gd) in patients with MD, and this technique was gradually established for MD diagnosis. However, few studies reported their diagnostic sensitivity and specificity in clinical application. This prospective study aimed at investigating the clinical characteristics and magnetic resonance imaging (MRI) results of patients with MD, and analyzing the relationship between clinical results and MRI findings in MD patients. Our study shows that the diagnostic sensitivity and specificity of MRI were 79.2% and 80.7% respectively. Moreover, there was no significant correlation between hearing levels and cochlear grading scores, nor vestibular grading scores. The duration of disease was not significantly associated with cochlear or vestibular grading scores. These findings suggest that IT-Gd MRI offers reliable radiological diagnostic criteria for MD.

## Introduction

Ménière disease (MD) is an idiopathic inner ear disorder characterized by recurrent episodic vertigo, fluctuating hearing loss, tinnitus and aural fullness^1^, and endolymphatic hydrops (EH) is considered to be its pathological basis. The reported prevalence of MD varies from 190 to 270 per 100,000 inhabitants^2,3^, due to the uncertainty of its diagnostic criteria, as well as the differences in research methodology and population surveyed. According to present diagnostic criteria, the ‘definite’ diagnosis of MD is based on the patients’ medical history and audiological test result. Other examinations, including a battery of electrophysiological, and vestibular function tests, such as the cochlear electrogram and caloric test are supplementary examinations for diagnosing MD. However, none of these tests can be deemed to be the diagnostic gold standard of MD, making its definite diagnosis difficult.

In 2004, Duan et al.^4^ for the first time reported the use of magnetic resonance imaging (MRI) following round window gadolinium application in guinea pigs. The imaging showed a preferential enhancement of the perilymph, enabling the visualization of the membrane labyrinth and the perilymphatic compartment. The principle of this technology is that after IT-Gd, the contrast agent can penetrate through the round window and the oval window membrane distributing mainly into perilymphatic space rather than endolymphatic space, thus causing a negative contrast in the non-enhancing endolymphatic spaces. If EH exists, the negative-signal endolymphatic space will become distended, and can be identified by evaluation of the filling defect volume in MRI.

This finding enlightened researchers that MRI may be feasible to evaluate the EH of MD patients. Until 2007, when Nakashima^5^ visualized EH using MRI by intratympanic gadolinium-based contrast media (GBCM) administration (IT-Gd) in patients with MD, this technique was gradually established for MD diagnosis. Although IT-Gd is off-label usage of gadolinium, it is widely accepted in China and several Chinese hospitals have applied this technology.

Since IT-Gd MRI has been used in multiple research institutions, various methods of EH imaging, contrast material administration and evaluation criteria for EH have been proposed. However, few studies reported their diagnostic sensitivity and specificity in clinical application. Since 2015, we have performed IT-Gd MRI on patients who were diagnosed with definite MD, and investigated their clinical characteristics and MRI results.

## Methods

### Participants

We performed a prospective study with 117 patients who were referred to our hospital between July 2015 and December 2017. All patients had a diagnosis of definite MD. The diagnostic criteria were based on the latest guidelines revised in 2015^1^. Exclusion criteria were as follows: (1) conductive or mixed hearing loss; (2) middle ear infections; (3) vestibular schwannoma; (4) endolymphatic sac tumor; (5) other diseases which can independently explain the clinical symptoms of vertigo or hearing loss, including transient ischemic attack, sudden hearing loss, otosclerosis, vestibular migraine and benign paroxysmal positional vertigo, etc.

The study was carried out with approved ethical permission by the Second Affiliated Hospital of Nanchang University Institutional Review Board. Written, informed consent was obtained from all patients. All methods were carried out in accordance with relevant guidelines and regulations.

### Test procedure

All patients underwent a detailed clinical interview. Clinical data and past medical history were obtained. Routine general physical examination, detailed neuro-otological examination, routine audiological and otoendoscopy were also performed. To examine whether an endolymphatic hydrops of the inner ear existed we requested an IT-Gd MRI for every patient included in the study. Caloric testing was conducted in 29 of the 117 patients due to lack of equipment at the beginning of the study.

### Hearing evaluation

All patients’ hearing was evaluated with pure tone audiogram (ASTERA 1066, Otometrics, Copenhagen, Denmark) and tympanometry (Otoflex 100, Otometrics, Copenhagen, Denmark), and all hearing tests were performed by the same audiologist. Air and bone conduction were assessed at frequencies of 250 Hz, 500 Hz, 1 kHz, 2 kHz, 4 kHz, and 8 kHz.

Pure tone average (PTA) was calculated as the mean of air conduction thresholds at 0.5, 1, 2, and 4 kHz^6^. The hearing loss levels were categorized into five grades: mild (26–40 dB HL), moderate (41–55 dB HL), moderate to severe (56–70 dB HL), severe (71–90 dB HL), and profound (> 90 dB HL)^7^.

### Vestibular tests procedure

The caloric test was conducted in 29 patients. After air caloric irrigation of the ear canal with warm (50 °C) and subsequently cooled air (24 °C) for 60 s, evoked nystagmus was recorded by videooculographic recording techniques (VNG ULMER, SYNAPSYS, Marseille, France), unilateral weakness (UW) and directional preponderance (DP) were measured. The positive caloric test result was defined as UW ≥ 20%, and DP ≥ 20%, respectively.

### MRI protocol

#### IT-Gd procedure

All patients underwent IT-Gd bilaterally 24 h before MRI scan. The procedure was as follows: the patient first lay in lateral position with the ear to be injected upwards. After topical anesthesia with 1% tetracaine, 0.5 ml gadopentetate glucosamine (Bayer-Schering Pharma, Berlin, Germany) diluted 1:8 with physiological saline, was injected into the middle ear after puncture of posteriorinferior part of tympanic membrane. Then, the patient remained still for about 30 min after injection. Afterwards, the patient lay on the opposite side, and underwent IT-Gd contralaterally in the same way. 24 h after IT-Gd, MRI was performed.

#### MRI scan technique

Imaging examinations were carried out on a 3 T MRI scanner (HDX 3.0 T MR, General Electric, USA) with a 8-channel SENSE head coil. Three-dimensional fluid attenuated inversion recovery (3D-FLAIR) sequence with the CUBE technique was performed 24 h after IT-Gd. The imaging parameters are listed in Table 1.

**Table 1:** Parameters of magnetic resonance imaging scan.

	3D-FLAIR
Repetition time (TR)	6000 ms
Echo time (TE)	123.5 ms
Inversion time (TI)	1868 ms
Flip angle (FA)	Variable, average value: 90°
Echo train length (ETL)	140
Field of view (FOV)	24 cm × 16 cm
Matrix size (MS)	320 × 320
Slice thickness	1.2 mm
Gap	0.5 mm
Scan time	5′39
Bandwidth	31.25

#### Measuring and grading method

**Figure 1:**
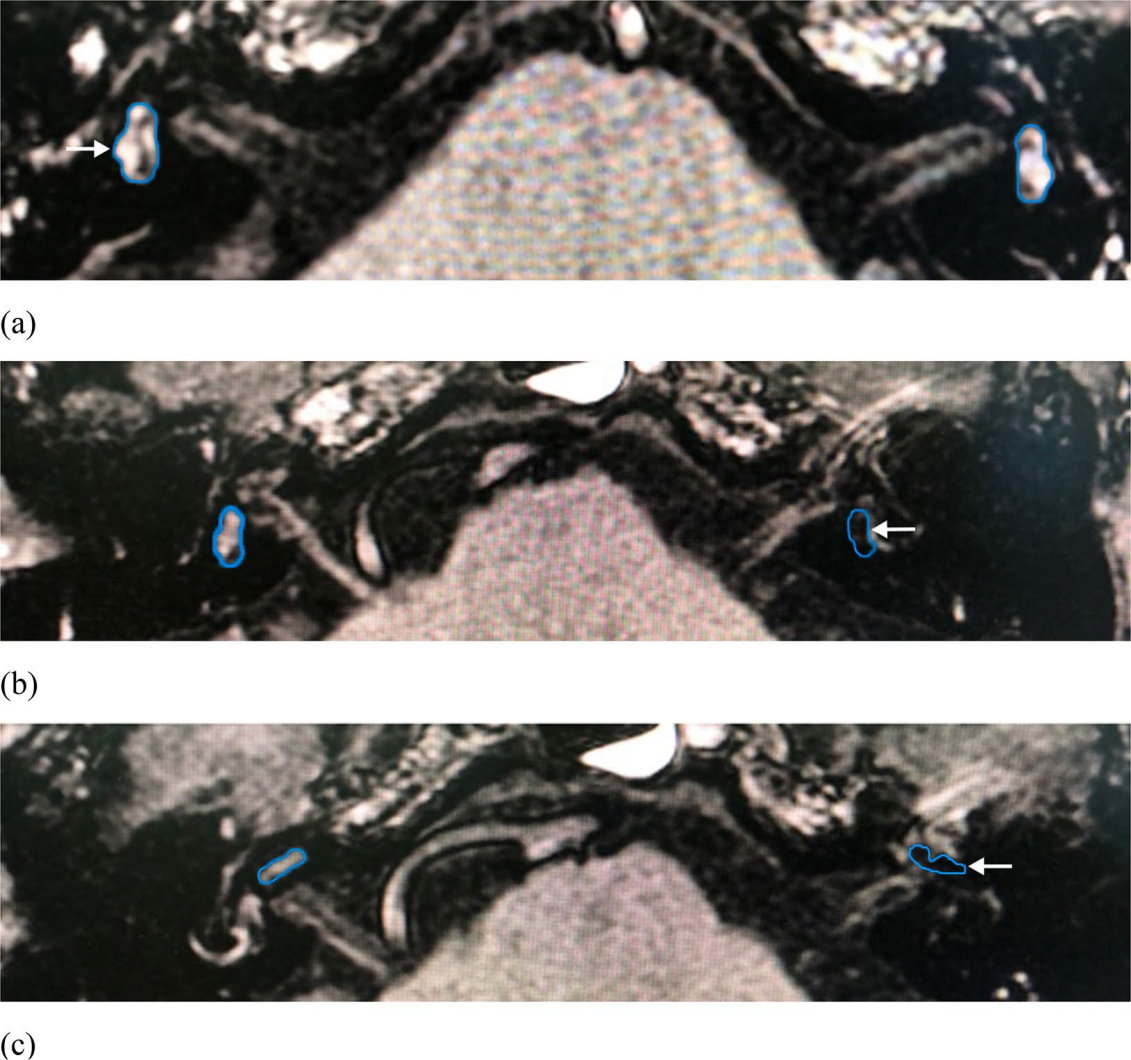
Three-dimensional fluid attenuated inversion recovery (3D-FLAIR) images of patients who diagnosed with definite MD. (**a**) The region of interest (ROI) placement for the mild endolymphatic hydrops (grade 1) in right-sided vestibule (white arrow). (**b**) The ROI placement for the obvious endolymphatic hydrops (grade 2) in left-sided vestibule (white arrow). (**c**) The ROI placement for the significant endolymphatic hydrops (grade 2) in left-sided cochlea (white arrow).

Referring to the measuring and grading method reported by Nakashima^8^, images were evaluated independently by two radiologists who were blind to the clinical data. If the two radiologists had different opinions regarding the grading score, they would discuss the results to reach a consensus. EH was classified into three grades: none, mild and significant (grade 0–2). The presence of EH is characterized by distended negative-signal endolymphatic spaces within the labyrinth. The cochlea and vestibule are graded separately as follows: in the cochlea, EH is deemed absent (Grade 0) if there is no bulging of the cochlear duct (CD) and the CD is slit-shaped or triangular. In grade 1 cochlear EH, the non-enhancing cochlear duct dilates into the scala vestibuli, partially obstructing the scala vestibuli. Grade 2 cochlear EH is defined as the endolymph of the CD is larger than that of the scala vestibuli. In the vestibule, if the endolymphatic area is less than 1/3 of the total vestibular area, there is no EH (grade 0). Grade 2 vestibular EH is defined as the endolymphatic area of the vestibule is more than one half of the total area. Grade 1 EH is intermediate between the two (Fig. 1).

### Follow-up procedure

All patients were followed at 1 week, 1, 3 and 6 months after the examination. Their clinical symptoms were reviewed, and general otorhinolaryngological examination, otoendoscopy and pure tone audiogram were performed in all subjects at each follow-up visit.

### Statistical analysis

Descriptive statistical analysis was employed for data analysis, including mean, minimum and maximum. The diagnostic sensitivity and specificity of MRI were analyzed using cross-tabulation. Linear regression was carried out to analyze the relationship between the hearing levels the clinically affected ear and the duration of the disease. Spearman correlation analysis was conducted to explore the correlation between the hearing outcomes and the grading scores of EH in the cochlea and vestibule, as well as the relationship between the duration of MD and the degrees of EH in the cochlea and vestibule. All analyses were conducted using SPSS version 25 for Windows.

## Results

### Clinical characteristics

**Figure 2:**
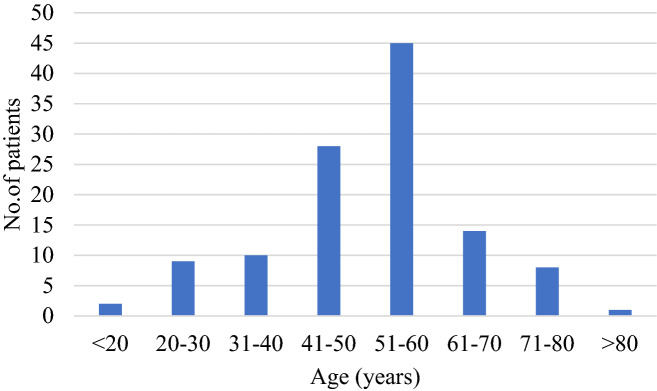
Age distribution of first onset in MD.

A group of 117 patients (50 males and 67 females) were included in our study, with a male to female ratio of 1/1.34. Among all patients, 108 patients suffered from unilateral MD and 9 had bilateral MD. The average age of all patients when they visited our hospital was 56 years (range 20–81 years). The average age at onset of the first MD episode according to the patient’s medical history was 51 years (range 13–81 years), with a peak prevalence in the group of patients 51–60 years of age (Fig. 2). The duration of MD ranged from 20 days to 16 years; the average duration of disease was 4 years and 11 months. No patients had autoimmune diseases comorbidities, a family history of MD or migraine.

### Audiological characteristics

**Figure 3:**
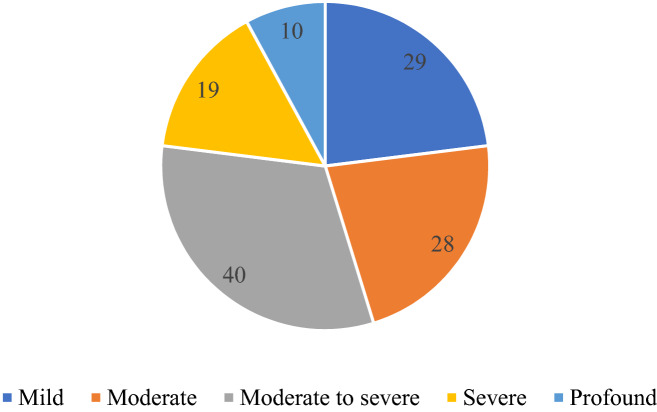
The numbers of affected ears (126 ears) in different hearing level group.

The PTA of all affected ears (126 ears) was 25–120 dB HL, and the average PTA was 60.6 dB HL. The hearing loss in 29 (23.0%) ears was classified as mild, in 28 (22.2%) as moderate, in 40 (31.7%) as moderate to severe, in 19 (15.1%) as severe, and in 10 (7.9%) as profound hearing loss (Fig. 3). Tympanogram was type A in all patients. There was no correlation between hearing levels of the clinically affected ear and the duration of MD (p = 0.185).

### Vestibular test results

All the 29 patients who underwent caloric test suffered from unilateral MD, 13 (44.8%) patients presented horizontal semicircular canal paresis in the diseased side. Abnormal DP results were observed in 19 (65.5%) patients.

### Magnetic resonance imaging results

Among all 234 ears (including affected and unaffected ears), the MRI results of 187 ears were in good agreement with the clinical diagnosis, and 47 ears were not. The diagnostic sensitivity and specificity of MRI were 79.2% and 80.7% respectively (Table 2). Both the MRI results and clinical complaints were positive in 99 ears, their grading scores of cochlear and vestibule results are shown in Table 3. There were 26 cases with false negative MRI results and the average duration of disease in these patients was 4 years and 5 months. On the other hand, 21 cases had false positive MRI results, and these patients’ average duration of disease was 4 years and 4 months. There was no significant correlation between hearing levels and cochlear grading scores (p = 0.407), nor vestibular grading scores (p = 0.479). The duration of disease was not significantly associated with cochlear scores (p = 0.247) or vestibular grading scores (p = 0.712).

**Table 2:** Cases of positive and negative results of clinic diagnose and IT-Gd MRI among all ears (234 ears).

	Guideline diagnostic criteria	
	Positive	Negative
**IT-Gd MRI**		
Positive	99	21
Negative	26	88

**Table 3:** The numbers of ears in different cochlear and vestibular scores (n = 99 ears, n (%)).

Structures	Gradings		
	0	1	2
Cochlear	8 (8)	52 (52.5)	39 (39.4)
Vestibule	1 (1)	31 (31.3)	67 (67.7)

### Follow-up results

All patients’ ruptured eardrums healed within 1 week after puncture without treatment. No local or systemic adverse reactions such as hearing loss, vertigo or allergic reaction occurred during 1 week after IT-Gd. During the later follow-up period, 25 cases suffered from frequent onset of hearing loss and vertigo, attributing to their MD; No other adverse reactions occurred.

## Discussion

Our study shows that the male to female ratio among patients was 1/1.34, which corroborates previous findings that this ratio was 1/1.3, 1/1.57 and 1/1.189, respectively^2,9,10^. It is hypothesized that the slight female preponderance may be due to hormonal influence. In addition, our study shows that MD is prevalent among middle-aged patients, due to the peak age incidence was in the group 51–60 years of age. Studies have shown that a definitive association between migraine and MD, and the reported incidence of migraine in MD patients varied from 4.5 to 56%^11^. However, in our study, no patients presented headache, as well as other migraine symptoms, such as visual symptoms, photophobia or phonophobia during the vertigo attack or intermission. We speculated that because most patients who presented migraine symptoms may firstly visited the department of Neurology in our hospital and received treatment there, which may be able to explain the variation of the migraine prevalence between our study and previous studies.

A previous study demonstrated that the diagnostic sensitivity and specificity of MRI by IT-Gd was 97.5% and 98%, respectively^12^. Another study showed that the sensitivity varied from 65.4 to 84.6%, and the specificity ranged from 92.3 to 97.4%^13^, but they used different grading methods from ours. By contrast, our diagnostic sensitivity and specificity MRI results were 79.2% and 80.7%, respectively. One possible explanation for the discrepancy between our study and other studies is that the border between the cochlear duct and surrounding bone is not so clear, making manual tracing of the cochlear duct difficult, consequently causing operator bias. Another limitation of this semi-quantitative grading method is that saccular and utricular hydrops are not assessed in this system, as the saccule was reported to be the most specifically involved structure in MD, and saccular hydrops was proven to be significantly associated with the degree of low to medium hearing loss^14^. Although the diagnostic value of MRI in our study is not as high as previous studies reported, we consider MRI a valuable tool for the diagnosis of MD, since there are no other tests can be used for direct EH visualization.

We found that the MRI results were consistent to the clinical diagnosis in the majority of cases. However, a weaker enhancement effect within the perilymphatic space may also be visualized in ‘unaffected’ clinically healthy ears, which can cause an inconsistency between the clinical diagnosis and the MRI findings. A possible explanation is that EH might be systematic and thus bilateral as reported by some clinical studies and histopathological examination of temporal bones in autopsies. This indicates that the clinically unaffected ear may be not completely ‘healthy’ in some patients^15–17^. This may explain our study’s result that 21 patients’ clinically non-affected ear also presented positive result in MRI. These patients may develop to bilateral hearing loss and vestibular hypofunction, and long-time follow-up is needed. In our study, 26 cases presented with false negative MRI results, one possible reason is that EH may not directly account for its symptoms, because as it is reported that some patients with EH but did not exhibit MD symptoms^18^.

Previous middle ear diseases can also affect the permeability of the round and oval window membrane and block the contrast agent from penetrating into the inner ear. This can lead to false positive EH pictures in the otherwise clinically healthy ears with no established MD. In order to avoid this situation, prior to the IT-Gd injection, we used otoendoscopy to exclude tympanic membrane changes usually accompanying middle ear problems e.g. calcified, thickened tympanic membrane or healed tympanic membrane perforation. However, it may still be that some patients with previous pathological conditions presented normally in otoendoscopy.

Our study demonstrates that there is no correlation between hearing levels and the degree of EH in cochlear and vestibule, which is inconsistent with some studies^19,20^. However, Gürkov et al. pointed out the association between clinical symptoms and EH is not uniform in each patient, as hearing can be preserved despite prominent EH^21^. The reason for this lack of correlation is unknown. More clinical data needed to investigate these associations in MD patients. In addition, there was no correlation between the duration of the disease and the hearing levels, or the degree of EH in cochlear and vestibule. These findings indicate that the progression of the disease is not consistent with the severity of disease, some patients may develop into severe disease in a short time, whereas some patients’ disease may progress slowly.

Although most institutions adopt the IT-Gd method to diagnose EH, intravenous gadolinium-based contrast media administration (IV-Gd) method is also increasingly applied. This new approach enables the evaluation of the perilymphatic space size bilaterally without GBCM injection in asymptomatic ears. A previous study has revealed that after a single IV-Gd dose, preferential perilymphatic enhancement can be achieved with 3D-FLAIR sequences and a 3 T MRI in human volunteers, but GBCM cannot be distributed into the endolymph. Other studies have used a double or triple dose of IV-Gd in human volunteers and reported the visualization of EH^22–24^. However, it was difficult to precisely define the scala media^24^. In addition, overuse of IV-Gd might cause nephrogenic systemic fibrosis in patients with impaired renal function. Some researchers utilized an alternative eustachian tube approach to deliver GBCM^25^. This method was described as a non-invasive procedure. However, most institutions still use trans-tympanic-membrane-puncture as the routine route for GBCM administration, due to the technical simplicity.

Our study supports previous findings about the safety of the IT-Gd method. A previous study found that after an injection of an eightfold diluted GBAB, the concentration of GBCM within the perilymph was estimated to be 1.0 × 10^−4^ mol/L, which was a 5000-fold dilution of the original solution^26^. Therefore, the damage of the contrast medium to the inner ear hair cells is very limited. This result was also corroborated by another prospective study, which observed no significant hearing deterioration 1 week after IT-Gd^27^. Furthermore, Duan et al. found that local GD application did not change ABR thresholds^27^. Other possible adverse effects, such as tympanic membrane perforation, did not occur in our study either. However, it should be noted that the usage of IT-Gd is off-label; the potential long-term adverse effects of IT-Gd still require further study.

## Conclusion

Considering the high sensitivity and specificity, IT-Gd MRI offers reliable imaging diagnostic criteria for MD.

## Data Availability

All data generated or analyzed during this study are included in this published article.
